# Regression models for interval censored data using parametric pseudo-observations

**DOI:** 10.1186/s12874-021-01227-8

**Published:** 2021-02-15

**Authors:** Martin Nygård Johansen, Søren Lundbye-Christensen, Jacob Moesgaard Larsen, Erik Thorlund Parner

**Affiliations:** 1 Unit of Clinical Biostatistics, Aalborg University Hospital, Sdr Skovvej 15, Aalborg, 9000 Denmark; 2 Department of Clinical Medicine, Aalborg University, Aalborg, Denmark; 3 Department of Cardiology, Aalborg University Hospital, Aalborg, Denmark; 4 Section for Biostatistics, Department of Public Health, Aarhus University, Aarhus, Denmark

**Keywords:** Pseudo-observations, Interval censoring, Flexible parametric model

## Abstract

**Background:**

Time-to-event data that is subject to interval censoring is common in the practice of medical research and versatile statistical methods for estimating associations in such settings have been limited. For right censored data, non-parametric pseudo-observations have been proposed as a basis for regression modeling with the possibility to use different association measures. In this article, we propose a method for calculating pseudo-observations for interval censored data.

**Methods:**

We develop an extension of a recently developed set of parametric pseudo-observations based on a spline-based flexible parametric estimator. The inherent competing risk issue with an interval censored event of interest necessitates the use of an illness-death model, and we formulate our method within this framework. To evaluate the empirical properties of the proposed method, we perform a simulation study and calculate pseudo-observations based on our method as well as alternative approaches. We also present an analysis of a real dataset on patients with implantable cardioverter-defibrillators who are monitored for the occurrence of a particular type of device failures by routine follow-up examinations. In this dataset, we have information on exact event times as well as the interval censored data, so we can compare analyses of pseudo-observations based on the interval censored data to those obtained using the non-parametric pseudo-observations for right censored data.

**Results:**

Our simulations show that the proposed method for calculating pseudo-observations provides unbiased estimates of the cumulative incidence function as well as associations with exposure variables with appropriate coverage probabilities. The analysis of the real dataset also suggests that our method provides estimates which are in agreement with estimates obtained from the right censored data.

**Conclusions:**

The proposed method for calculating pseudo-observations based on the flexible parametric approach provides a versatile solution to the specific challenges that arise with interval censored data. This solution allows regression modeling using a range of different association measures.

**Supplementary Information:**

The online version contains supplementary material available at (10.1186/s12874-021-01227-8).

## Background

In medical research, the outcome is often an event such as death, occurrence of a disease, or a treatment-related event during a follow-up period. Some individuals will be event-free throughout follow-up, but the event may occur after the end of follow-up. This kind of incomplete follow-up is called *right censoring* and methods for dealing with this form of censoring are used very frequently in the medical literature. Right censored data thus consist of a mixture of exactly observed event times and censoring times. In other situations, the exact event times are never observed and the event status is only evaluated at certain time points, *examination times*, and the data are then said to be *interval censored*. This phenomenon occurs frequently when for example a specific group of individuals is monitored by routine examinations for a medical condition. In such cases, event times are known only to lie within a time interval from the last examination without the event to the first examination after the event has occurred. In practice, data can also consist of a mixture of right and interval censored data, e.g. when data are gathered from different sources. A standard assumption when analyzing interval censored data is that the examination times are independent of the event risk. In that case one can in the analysis ignore the distribution of the examination times, and treat the examination times as fixed. We will also assume that the examination times are independent of the event risk.

Interval censoring has posed a challenge to the medical research community that has proven hard to overcome and as a consequence it has become quite common to impute an event date using either the midpoint of the interval or the time of the first examination time where the event is observed to have occurred. Regression models for interval censored data has traditionally mostly been concerned with basic parametric regression models where inference can be performed by standard maximum likelihood methods and in which the estimators converge at a rate of $\sqrt {n}$. Parametric models are easily fitted using most common statistical software but each distributional family imposes rather strict assumptions on the shape of the hazard function and it is our impression that their use in applications has diminished in recent years; most likely due to reluctance to impose such assumptions, although covariate adjustment is straightforward in parametric models. A parametric approach that can accomodate different distributional characteristics is the piece-wise exponential proportional hazards model or equivalently a Poisson log-linear model where the hazard is assumed constant in some set of intervals of the follow-up time [1]. When events are plentiful the follow-up intervals can be made small enough to give a reasonable fit to practically any shape of the hazard function but when the data is more sparse with few events or the hazard has a more complex shape during follow-up the piece-wise exponential model has obvious limitations [2].

As an example of an interval censored dataset, we consider a group of patients with an implantable cardioverter-defibrillator (ICD), which is a kind of pacemaker that can protect against slow heart rhythm but also fast arrhythmias, which otherwise can result in hemodynamic compromise with loss of consciousness and cardiac arrest. The fast arrhythmias can be treated by fast pacing or delivery of a high voltage shock that restores the heart rhythm to normal. The ICD is placed in the subcutaneous tissue on the front of the chest below the left collarbone and is connected to the inside of the heart through a large blood vessel. The ICD lead gives the ICD the ability to continuously monitor the heart rhythm and if needed deliver the high voltage shock inside the heart. The ICD lead is the most sensitive part of an ICD system and is the part with the highest risk of failure either due to insulation failures or conductor fractures. The particular lead investigated is prone to a rather unique type of insulation failure because of a design flaw where the inner conductors over time work their way through the outer insulation. Such outer insulation failures, called *externalizations*, may be electrically silent at normal ICD follow-up and require dedicated fluoroscopic/X-ray imaging to be detected. The ICD is at risk of failing from such externalization events throughout follow-up, but patients can also have their ICD leads removed (extracted) for other reasons during follow-up, which obviously precludes an externalization event. We consider externalization as the event of interest and we are interested in estimating the association between the amount of slack in the lead body inside the heart and the time to externalization, since more lead slack puts the continuously moving lead body under more physical stress. In this setting, we have a combined competing risk of death or extraction of the ICD leads. To assess the association between lead slack and externalization, we are interested in comparing the cumulative risk of externalization at one or more time points.

In this application, interest lies in assessing the effect of the exposure on the cumulative risk of developing the outcome in the presence of the competing risks but existing methods are not well-equipped for this type of situation. However, in the right censored competing risk setting, *pseudo-observations* have been proposed [3] as a modeling approach which enables effect estimation on a number of different scales other than the hazard scale such as the cumulative incidence scale. This method is based on a transformation of the potentially censored time-to-event data into a set of complete data on which regression can be performed using generalized linear models to estimate the relevant effect parameters. When the aim is to model some function of the cumulative incidence, the transformation is based on the non-parametric Aalen-Johansen estimator of the cumulative incidence function.

A non-parametric estimator of the survival function based on interval censored data has been proposed by both Peto and Turnbull [4, 5]. The resulting Peto-Turnbull estimator is a piece-wise constant curve with relatively few jumps. A natural way to apply the pseudo-observation approach to interval censored data therefore seems to be to perform a transformation of the data based on the Peto-Turnbull estimator similarly to the pseudo-observation approach based on the Aalen-Johansen estimator. This approach has been investigated by Kim and Kim [6] in a competing risk setting. However, the asymptotic properties of the resulting pseudo-observations are unclear since the theory for pseudo-observations has been developed only for estimators with parametric $\sqrt {n}$ convergence rate [7], whereas the Peto-Turnbull estimator has slower *n*^1/3^ convergence rate [8].

Royston and Parmar [9] have proposed a *flexible parametric model* which is applicable to both right censored and interval censored data. This is a regression modeling framework where the log cumulative hazard function is estimated using a restricted cubic spline in log time. In the most simple form with no covariates this approach provides a way to model the cumulative incidence function and when covariates are included the model can be formulated as either a proportional hazards or a proportional odds model.

As in our example above, the event of interest in interval censored data is often a non-fatal event, so methods for handling interval censoring should accomodate death as a competing risk. For the remainder of this article, we consider only competing events for which the event time is exactly observed and refer to competing events as death for ease of terminology. In a competing risk setting with a right censored event of interest, we can model the cause-specific hazard functions separately by considering only the time to whatever event occurs first. But when the event of interest is interval censored, we are only observing the event if there is an examination after the event has occurred but before the individual is censored or dies. Hence, there might be some events of interest which are unobserved in the data. Because of this circumstance, the inference needs to take into account that the event of interest might or might not have occurred in the interval between the last examination time without the event of interest and time of death or censoring. To accomodate this, the data could be considered in an illness-death model [10] where the risk of death is also modeled after an event of interest has occurred.

Recently, an elegant approach to calculating pseudo-observations for interval censored data was proposed by Sabathé et al. [11] specifically for an illness-death model. This approach is based on modeling the three transition intensities either based on Weibull distributions or using M-splines and applying a penalized likelihood approach where more roughly shaped intensity functions are penalized using the second derivatives of the three M-splines squared. If the penalization parameters are all set to zero, the method simplifies to a full likelihood approach. This requires a high number of coefficients for each of the three splines depending on the order and the number of knots of the spline as well as three penalization parameters to be chosen by the analyst. Due to this high number of parameters, the authors do not recommend using their method in place of the traditional non-parametric pseudo-observation approach for data without interval censoring.

For right censored competing risk data, we have recently shown that in some situations calculating *parametric pseudo-observations* based on a marginal flexible parametric estimator of the cumulative incidence function can provide less variability in the effect estimates than that of traditional non-parametric pseudo-observations [12].

In this article, we propose an extension of this approach that applies to the interval censored setting and is targeted directly at estimating associations between an exposure and the event of interest. In the Proposed method section, we describe the proposed method in more detail and in the Simulation studies section we describe a simulation study that compares our proposed method to the existing methods. We present the results of these simulations in the Simulation studies section and present an analysis of the example data in the Application to ICD data section. We conclude the article with a discussion and conclusion in the Discussion section and Conclusion section.

## Methods

### Proposed method

We now give details on how the parametric pseudo-observation approach can be extended to cover interval censored settings with competing risks using an illness-death model.

An illness-death model involves an event of interest and the competing event death which gives three different states; 0 where neither event has occurred, 1 where only the event of interest has occurred, and 2 which is death with or without having experienced the event of interest. In the following, we will assume that all individuals are initially in state 0 at time *t*=0 and we let *h*_*kl*_ denote the hazard function describing transition from one state, *k*, to another, *l* and similarly we let *H*_*kl*_ denote the cumulative hazard function. To estimate the cumulative incidence function of the event of interest, *F*_01_(·), we will use the estimates of the transition-specific hazard functions and the relationship between these and the transition-specific cumulative incidence function,

1 $$\begin{array}{*{20}l} F_{01}(t) &= \int_{0}^{t} h_{01}(u) S(u) du, \end{array} $$

where *S*(·) is the event-free survival function defined as

$$\begin{array}{*{20}l} S(t) &= \exp \Bigl(-H_{01}(t)-H_{02}(t) \Bigr). \end{array} $$

Following the flexible parametric approach of Royston and Parmar [9] that generalizes a Weibull model, we estimate the transition-specific hazard functions by modeling the transition-specific log cumulative hazard functions using restricted cubic splines in *x*= ln(*t*). According to Royston and Parmar [9], a natural cubic spline with *m* internal knots, *ξ*_1_,…,*ξ*_*m*_, and external knots *ξ*_*min*_,*ξ*_*max*_ can be expressed as

$$\begin{array}{*{20}l} s(x;\boldsymbol{\gamma})=\gamma_{0} + \gamma_{1} x + \gamma_{2} v_{1}(x) + \cdots + \gamma_{m+1} v_{m}(x), \end{array} $$

where $v_{j}(x)=(x-\xi _{j})_{+}^{3} - \lambda _{j}(x-\xi _{min})_{+}^{3} - (1-\lambda _{j})(x-\xi _{max})_{+}^{3}$.Hence, we are assuming the model

$$\begin{array}{*{20}l} \ln\left(H_{kl}(t)\right) &= s_{kl}(x;\boldsymbol{\gamma}_{kl})  \\ &= \gamma_{kl,0} + \gamma_{kl,1} x + \gamma_{kl,2} v_{kl,1}(x) + \cdots \\&+ \gamma_{kl,m+1} v_{kl,m}(x), \end{array} $$

for going from state *k* to state *l*. For simplicity, we assume that the number of knots is *m* for all three splines. The model, hence, contains *m*+2 spline coefficients, ***γ***_*kl*_=*γ*_*kl*,0_,…,*γ*_*kl*,*m*+1_, for each transistion and corresponding spline knots *ξ*_*kl*,*min*_,*ξ*_*kl*,1_,…,*ξ*_*kl*,*m*_,*ξ*_*kl*,*max*_. Based on the spline coefficients, ***γ***_01_,***γ***_02_, and ***γ***_12_, we can express the transition-specific hazard function as

$$\begin{array}{*{20}l} h_{kl}(t) &= \frac{ds_{kl}(x;\boldsymbol{\gamma}_{kl})}{dt} \cdot \exp\left(s_{kl}\left(x;\boldsymbol{\gamma}_{kl}\right)\right)  \\ &= \frac{1}{t} \cdot \frac{ds_{kl}(x;\boldsymbol{\gamma}_{kl})}{dx} \cdot \exp(s_{kl}(x;\boldsymbol{\gamma}_{kl})). \end{array} $$

The derivative of *s*_*kl*_(*x*;***γ***_*kl*_) is

$$\begin{array}{*{20}l} \frac{ds_{kl}(x;\boldsymbol{\gamma}_{kl})}{dx} = \gamma_{kl,1} &+ \sum_{j=2}^{m} \left\{ \gamma_{kl,j} \cdot \left(3 (x - \xi_{kl,j})_{+}^{2}  \right.\right.\\ &- 3 \lambda_{kl,j} (x-\xi_{kl,min})_{+}^{2} \\ &\left.\left.- 3 (x-\xi_{kl,max})_{+}^{2} \right) \right\}. \end{array} $$

We consider a setting where the time to the event of interest can either be observed exactly (right censored) or interval censored but the time of death is always observed exactly (right censored). Estimation of the spline coefficients is performed using maximum likelihood methods and the contributions to the likelihood function, *L*(***γ***_01_,***γ***_12_,***γ***_02_), take different forms according to the event trajectory of each individual. These trajectories are determined by the occurrence and timing of the event of interest and death as described by Touraine et al. [13]

#### Maximum likelihood estimation

The observed trajectory of an individual can be described by the observed event status and observation time for both the event of interest, (*d*_1_,*t*_1_), and death, (*d*_2_,*t*_2_), as well as a time of the last examination time without the event of interest if any such has occurred, *l*_1_. This last negative examination time might be at time *l*_1_=0 if no negative examinations have occurred. For individuals with an interval censored event of interest, the event of interest is then known to occur in the interval (*l*_1_,*t*_1_). For individuals with an event of interest for which the time is observed exactly, *l*_1_ is not defined and for individuals with right censored data but no event of interest, we let *l*_1_ denote the time point at which follow-up ends for that individual. We now describe the contributions to the likelihood function for each trajectory. For the *i*’th individual, we use the following notation. *d*_1*i*_ indicates an observed event of interest (either exactly observed or interval censored) *l*_1*i*_ is the last known negative time point (potentially at time zero) *t*_1*i*_ is the observation time for the event of interest (either the exact time or the first positive examination time) *d*_2*i*_ indicates a competing event (exactly observed) *t*_2*i*_ is the observation time for the competing event

For short, we will denote each individual’s contribution to the likelihood function as *L*_*i*_.

*Trajectories 1 and 4*

For an individual with the event of interest observed at time *t*_1*i*_ exactly, followed by death or censoring at time *t*_2*i*_, the contribution is

$$\begin{array}{*{20}l} L_{i} = S(t_{1i})h_{01}(t_{1i})\frac{\exp(-H_{12}(t_{2i}))}{\exp(-H_{12}(t_{1i}))}h_{12}(t_{2i})^{d_{2i}}. \end{array} $$

*Trajectories 2 and 5*

For an individual with an examination without the event of interest or right censoring of the event of interest at time *l*_1*i*_ followed by death or censoring at time *t*_2*i*_, the contribution is

$$\begin{aligned} L_{i} = S(t_{2i})h_{02}(t_{2i})^{d_{2i}} + \int_{l_{1i}}^{t_{2i}}S(u)h_{01}(u)\frac{\exp(-H_{12}(t_{2i}))}{\exp(-H_{12}(u))}h_{12}(t_{2i})^{d_{2i}}du. \end{aligned} $$

*Trajectories 3 and 6*

For an individual with an interval censored event of interest occurring between time *l*_1*i*_ and *t*_1*i*_ followed by a death or censoring at time *t*_2*i*_, the contribution is

$$\begin{array}{*{20}l} L_{i} = \int_{l_{1i}}^{t_{1i}}S(u)h_{01}(u)\frac{\exp(-H_{12}(t_{2i}))}{\exp(-H_{12}(u))}h_{12}(t_{2i})^{d_{2i}}du. \end{array} $$

The likelihood function obtained by multiplying the relevant contributions for each individual can be maximized numerically by using e.g. the Newton-Raphson algorithm. The contributions corresponding to each of the six trajectories are given in the Appendix [see Additional file 1].

#### Initial values

For likelihood maximization in practice, it is worth considering how to provide initial values for the parameter vector (***γ***_01_,***γ***_02_,***γ***_12_) in order to achieve convergence in as few iterations as possible. We propose the following approach using midpoints for interval censored events of interest.

Modeling the transition from state 0 to 1 can be done by fitting a flexible parametric model with the spline knots chosen for this transition and using the midpoints between *l*_1*i*_ and *t*_1*i*_ for interval censored events of interest. From this fitted model we can calculate a predicted survival function to estimate 1 minus the cumulative incidence of the event of interest. For each individual that has not had an observed event of interst, we can then estimate the probability that they had an unobserved event of interest in the interval between their last negative examination time, *l*_1*i*_, and their end of follow-up time, *t*_2*i*_, as the difference in predicted survival between these two time points. We can then randomly assign these individuals as having had or not having had an unobserved event of interst based on their individual probabilities and then temporarily consider some of them as if they had an event of interest at the midpoint of the interval from *l*_1*i*_ to *t*_2*i*_. This allows us to more accurately estimate the remaining two transitions.

The transitions from state 0 to 2 and from 1 to 2 can now be modeled, again using flexible parametric models with the relevant knots, using the updated event and status variables and imposing delayed entry at the time of the event of interest for the transition from state 1 to 2.

#### Parametric pseudo-observations for interval censored data

By maximizing the likelihood function described above, we obtain parameter estimates $(\hat {\boldsymbol {\gamma }}_{01},\hat {\boldsymbol {\gamma }}_{02},\hat {\boldsymbol {\gamma }}_{12})$ which can be used to form an estimate, $\hat {\theta }^{IC}$, of the cumulative incidence. Similarly, for each observation, *i*, we can obtain a leave-one-out estimate $\hat {\theta }_{(-i)}^{IC}$ based on all observations except the *i*’th with the same spline knots as for the full-sample estimate. We can then define a set of parametric pseudo-observations for interval censored data, $\theta _{1}^{IC},\ldots,\theta _{n}^{IC}$, as

2 $$\begin{array}{*{20}l} \theta_{i}^{IC} = n\hat{\theta}^{IC} - \left(n-1\right) \hat{\theta}_{(-i)}^{IC}, \quad \text{for \(i=1, \ldots, n\)}. \end{array} $$

The pseudo-observations thus defined can be analyzed using generalized linear models with a sandwich estimator of the variance in the same way as both non-parametric and parametric pseudo-observations for right censored data [3, 12]. Let *T*_1_ denote the observation time for the event of interest and *D*_1_ the corresponding event indicator. To estimate the risk difference or the relative risk at time *t* based on the suggested pseudo-observations and a covariate vector for the *i*’th individual ***Z***_*i*_=(*Z*_*i*1_,…,*Z*_*ip*_), we can then express the model as

$$\begin{array}{*{20}l} g \left(\mathrm{E}\left[\mathrm{I}(T_{1} \le t,D_{1}=1)\right] \right) = \beta_{0} + \sum_{j=1}^{p} \beta_{j} Z_{ij}, \end{array} $$

where *g*(·) is the relevant link function, *g*(*x*)=*x* for the risk difference and *g*(*x*)= ln(*x*) for the relative risk, and ***β***=(*β*_0_,…,*β*_*p*_) is the vector of regression coefficients. Estimates of the regression coefficients can be obtained solving the estimating equation

$$\begin{array}{*{20}l} \sum_{i=1}^{n} \left(\frac{\partial}{\partial \boldsymbol{\beta}} g^{-1}(\boldsymbol{\beta}^{\intercal}\boldsymbol{Z}_{i}) \right)^{\intercal} V_{i}^{-1} \left(\hat{\theta}_{i}^{IC} - g^{-1}(\boldsymbol{\beta}^{\intercal}\boldsymbol{Z}_{i}) \right) = 0, \end{array} $$

where *V*_*i*_ is a working covariance matrix that takes the correlation between pseudo-observations at different time points into account. In the case of just one time point, *V*_*i*_ is an estimate of the variance.

### Simulation studies

#### Data generation

We simulated datasets imposing a non-random binary exposure, *x*, such that half of the individuals are exposed and the other half is non-exposed and an administrative censoring at time *t*=5.

For the event of interest, we simulated realizations of a random variable *T*_01_∼Weibull(*k*_01_(*x*),*b*_01_(*x*)), with scale parameters *k*_01_(0)=0.06 and *k*_01_(1)=0.12 and shape parameters *b*_01_(0)=0.5 and *b*_01_(1)=0.4. These parameters were chosen to give approximately the same event rate that was observed in the ICD example already introduced. We simulated time to death from a random variable *T*_02_∼Exp(*λ*_02_) with intensity *λ*_02_=0.1. Based on these variables we define event indicators *δ*_01_ and *δ*_02_ according to which event occurs first if min(*T*_01_,*T*_02_)<5. Hence, all individuals enter the study at time *t*=0 in state 0.

For individuals who experience the event of interest, we simulate the transition from state 1 to state 2 as another random variable *T*_12_∼Exp(*λ*_12_) with *λ*_12_=0.4. The time-to-event for this transition is then *T*_01_+*T*_12_ with censoring at *t*=5 and the event indicator is *δ*_12_.

To mimic a practical setting with a mixture of right and interval censored data, we consider the event of interest for some individuals to be interval censored and for the others to be right censored. This allocation follows a Bernoulli distribution with probability parameter *p*_*ic*_ for being interval censored. For individuals with interval censoring of the event of interest, we simulate examination times with a mean interval length of *Δ* and a random error following a normal distribution with mean zero and variance *σ*^2^. We continue adding examinations until either the event of interest has occurred or the induvidual has died or has been censored following an iterative formula for examination times,

$$\begin{array}{*{20}l} e_{i+1} = e_{i} + \delta_{i}, \end{array} $$

where *δ*_*i*_∼*N*(*Δ*,*σ*^2^). This gives rise to the variable *l*_1*i*_ which is the last known time with a negative status for the event of interest and the variable *t*_1*i*_ which is the first known positive status. For individuals with an exactly observed event of interest, we let *l*_1*i*_=*t*_1*i*_ be the event time, and for right censored individuals in which we do not observe an event of interest will have *l*_*i*_=*t*_1*i*_=*t*_2*i*_ which is the time of death or censoring.

For the simulations, we performed 1 000 repetitions of datasets of sample size *n*=250, where *p*_*ic*_=80*%* of the events of interest are interval censored, and the mean time between examinations is *Δ*=1 with *σ*^2^=0.2.

#### Data analysis

In each dataset, we calculated three sets of pseudo-observations for the event of interest based on three different approaches.

- $\theta _{1}^{E},\ldots,\theta _{n}^{E}$ Potentially unobservable exact right censored event times for all individuals. These will serve as a way to measure the empirically highest achievable precision.
- $\theta _{1}^{IC},\ldots,\theta _{n}^{IC}$ Proposed method for taking interval censoring into account.
- $\theta _{1}^{S},\ldots,\theta _{n}^{S}$ Method for taking interval censoring into account proposed by Sabathé et al.

For each set of pseudo-observations we fitted the same generalized linear models with identity and log link functions to estimate the risk, risk difference, and relative risk of experiencing the event of interest before time *t*=3 and compared the resulting parameter estimates to true values which we obtained by calculating proportions of the event of interest in a simluated dataset with 10^8^ observations. If the estimation of spline coefficients for either the full sample or one or more leave-one-out subsamples did not converge or if the generalized linear regression model gave unreasonable estimates (cumulative incidence not in (0,1), risk difference not in (−1,1), relative risk not in (10^−1^,10)), we considered the results to be unvalid and ignore them in the following. Based on the obtained estimates, we then calculated the median bias, the empirical standard error (empSE) and the confidence interval coverage probability. We calculated confidence intervals for the cumulative incidence on a logarithmic scale to accomodate the non-symmetrical nature of the cumulative incidence scale. To compare the precision of the estimation between the methods, we calculated a relative empSE with the empSE of the $\theta _{i}^{E}$s as the reference value. We also calculated the root mean squared error (RMSE) and the average of the standard errors from the regression models (modSE) [14, 15].

We generated data and performed all pseudo-observation calculations except the $\theta _{i}^{S}$s as well as regression modeling using Stata/MP version 16.1. To calculate the $\theta _{i}^{S}$s we used R version 3.6.3 and the packages SmoothHazard and pseudoICD.

## Results

### Simulation studies

**Fig. 1:**
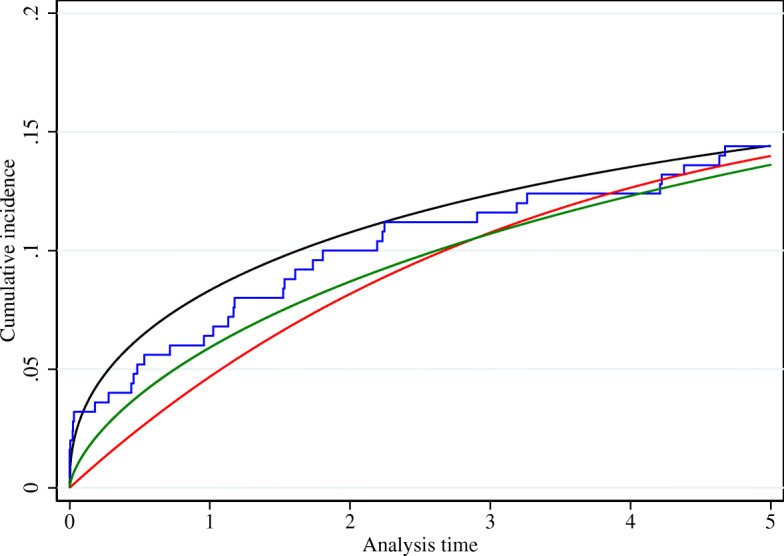
Full-sample estimators of the cumulative incidence function in one of the simulated datasets. Black curve: True cumulative incidence function. Blue curve: Aalen-Johansen estimator on exact event times. Red curve: Penalized likelihood estimator used in the approach by Sabathé et al. Green curve: Flexible parametric approach used in our proposed approach

To illustrate the three different estimation approaches, we have shown the full-sample estimators on which each of the compared approaches are based for a randomly chosen simulated dataset in Fig. 1. The black curve in the figure is the true cumulative incidence function based on the simulated dataset with 10^8^ observations. The blue curve is the Aalen-Johansen estimator based on the exactly observed event times, which shows that in this particular dataset, the cumulative incidence is slightly lower than expected. Both the penalized likelihood estimator (red curve) and the flexible parametric estimator (green curve) follow the estimator based on the exact event times reasonably well.

The results of the simulation study are shown in Table 1. In the 1 000 datasets, there were on average 146 events of interst but only 120 that we register when considering the data as interval censored. We focus mainly on the estimates of absolute cumulative incidence of the event of interest. The proposed method gave unvalid estimates in about 5% of the 1 000 datasets while the method proposed by Sabathé et al. never did.

**Table 1:** Results of the simulations in the general set-up based on estimation of cumulative incidence, risk difference and the logarithm of relative risk

Method	Bias	empSE	Relative empSE	modSE	RMSE	Coverage (95% CI)	Valid est.
Cumulative incidence (true value: 0.1236)							
Exact	-0.002	0.019	1 (ref.)	0.019	0.019	95.5 (94.0 to 96.6)	999
IC	-0.005	0.026	1.32	0.025	0.026	94.7 (93.1 to 96.0)	942
Sabathé et al.	-0.005	0.023	1.17	0.023	0.023	95.3 (93.8 to 96.5)	1000
Risk difference (true value: 0.0673)							
Exact	0.000	0.038	1 (ref.)	0.039	0.038	95.5 (94.0 to 96.6)	999
IC	-0.005	0.059	1.52	0.052	0.059	94.2 (92.6 to 95.5)	953
Sabathé et al.	-0.003	0.044	1.14	0.045	0.044	95.3 (93.8 to 96.5)	1000
Logarithm of relative risk (true value: 0.5590)							
Exact	0.010	0.348	1 (ref.)	0.341	0.349	96.2 (94.8 to 97.2)	999
IC	-0.025	0.456	1.31	0.454	0.456	95.8 (94.3 to 96.9)	944
Sabathé et al.	-0.014	0.414	1.19	0.409	0.415	96.6 (95.3 to 97.6)	1000

IC: The proposed method for calculating pseudo-observations for interval censored data

empSE: Empirical standard error, defined as standard deviation of parameter estimates

Relative empSE: Defined as empSE divided by empSE of parameter estimates using the exact method

modSE: Average model standard error

RMSE: Root mean squared error

CI: Confidence interval

Using the exactly observed data, the parametric pseudo-observations perform very well and we obtain virtually unbiased estimation of the true value of the cumulative incidence function at time *t*=3, which is 0.1236, with an empirical standard error of 0.019 and coverage probability close to the nominal value of 95%. Analysing the interval censored data using our proposed parametric pseudo-observations, we still get an unbiased estimator but the empirical standard error is 37% higher compared to using the exactly observed data due to the added uncertainty inherent in the interval censored data. The coverage of this method is also reasonably close to 95%. In terms of bias and coverage, the method proposed by Sabathé et al. performs quite similarly to our proposed method while the empirical standard error of the cumulative incidence estimates is slightly lower for the Sabathé et al. method. This might be explained by the additional three penalization parameters which control the smoothness of the fitted M-splines but must be provided explicitly or determined from the data using an approximate likelihood technique [13].

Estimating associations with the exposure gives small biases for both the risk difference and relative risk using either our proposed method or that of Sabathé et al. and the coverage probabilities are in good agreement with the nominal value.

### Application to ICD data

**Fig. 2:**
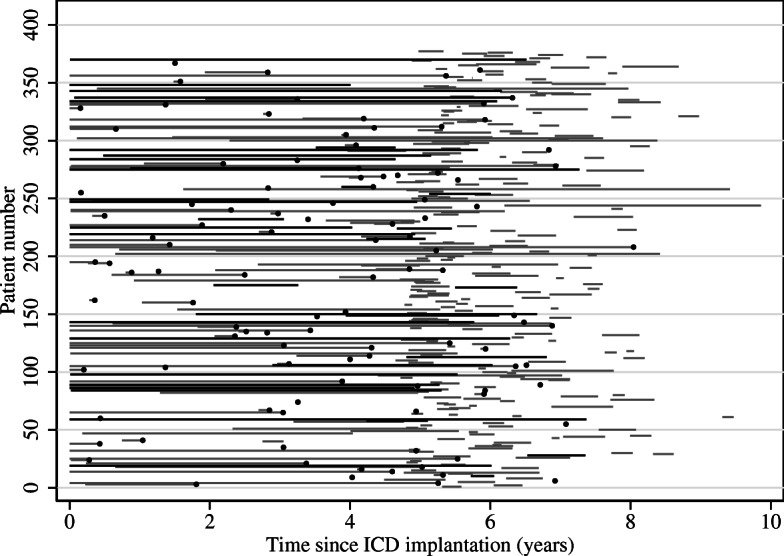
Visualization of the interval censored real example dataset. A black line indicates an interval with an observed externalization, a grey line indicates an interval with no observed externalization, black dots indicate deaths or lead extractions

Our ICD dataset holds data on 377 patients who are followed from the time of ICD implantation and for a maximum of about 10 years. During follow-up we have information on our event of interest, externalization status, at each fluoroscopic examination time and on the date of death or lead extraction if this occurred. The dataset, hence, consists only of interval censored data for the event of interest and right censored data for death or lead extraction. We show the trajectory for each patient in Fig. 2 where lines indicate an observation interval colored black for intervals ending at a positive examination and grey if we do not observe externalization and black dots indicate death or lead extraction times. We observed 37 externalization events and 106 cases of death or lead extraction during follow-up.

**Fig. 3:**
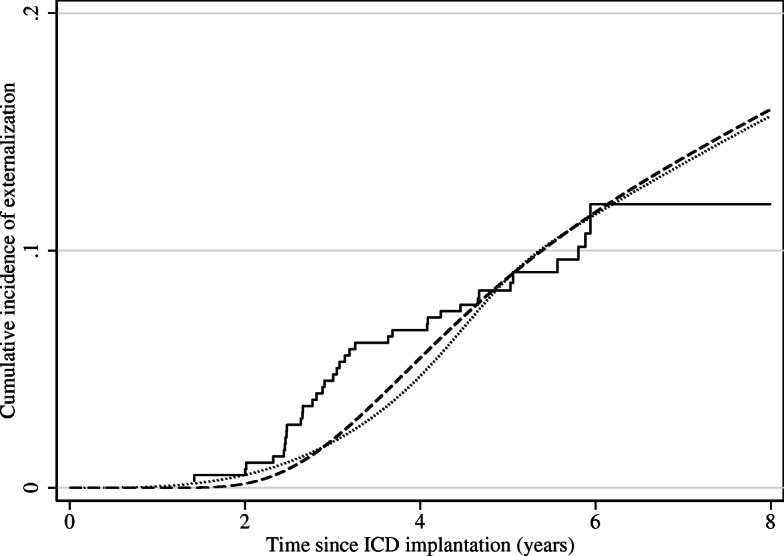
Estimated cumulative incidence of externalization. Solid curve: Aalen-Johansen estimator in a competing risk model. Dashed curve: Flexible parametric estimator with 3 knots based on an illness-death model fitted on the full sample. Dotted curve: Flexible parametric estimator with 4 knots based on an illness-death model fitted on the full sample

We first estimated the cumulative incidence function for the externalization event based on a competing risk model using the non-parametric Aalen-Johansen estimator [16] applied to the midpoints of the intervals. This is illustrated by the solid step function in Fig. 3. The dashed and dotted curves in the figure show the estimator based on the flexible parametric approach by fitting splines with 3 and 4 knots, respectively, to the interval censored data in an illness-death model. The three estimators seem to capture roughly the same shape of the cumulative incidence function although the Aalen-Johansen estimator based on midpoints shows a tendency to place the bulk of the events around 2–3 years due to a high number of patients having their first examination since implantation after roughly 5 years.

We then calculated parametric pseudo-observations for externalization events based on splines with both 3 and 4 knots evaluated at 5 years after ICD implantation and estimated the cumulative incidence at this time point as well as the risk difference and relative risk comparing patients with high lead slack to those with low lead slack. The results of the regression analyses are shown in Table 2. Using 3 knots for the splines, the estimated overall cumulative incidence of externalization within 5 years since ICD implantation is 8.6% (95% CI: [5.9 to 12.5]). In the crude analysis, patients with a high degree of lead slack have substantially increased externalization risk. In terms of absolute difference in risk, patients with high slack have a risk of externalization that is 9.2 percentage points (95% CI: [2.5 to 15.9]) higher than that of patients with low slack. On a relative scale, this difference corresponds roughly to a 3-fold increase in externalization risk with an estimated relative risk of 3.10 (95% CI: [1.41 to 6.78]). The association between lead slack and externalization risk might be confounded by factors such as the placement of the lead and the age of the patient, and to account for such potential confounding we have performed an analysis in which we adjust for lead placement (apical or septal) and age at implantation (above/below 65 years). Both the absolute and the relative adjusted estimates of association are quite similar to the crude estimates (see Table 2). If we use 4 knots instead of 3 for the splines, the estimated 5-year externalization risk is slightly lower at 8.2 (95% CI: [5.6 to 12.1]) and the estimates of the association with lead slack are rather consistent with the results based on 3 spline knots.

**Table 2:** Results of the generalized linear model regression analyses estimating the cumulative incidence, risk difference, and relative risk at 5 years since ICD implantation in the ICD dataset

Method	CIP (95% CI)	Risk difference (95% CI)		Relative risk (95% CI)	
		Crude	Adjusted	Crude	Adjusted
IC, 3 knots	8.6 (5.9*cc*12.5)	9.2 (2.5 to 15.9)	9.1 (2.4 to 15.9)	3.10 (1.41 to 6.78)	3.07 (1.20 to 7.86)
IC, 4 knots	8.2 (5.6 to 12.1)	8.9 (2.2 to 15.5)	8.8 (2.0 to 15.5)	3.13 (1.37 to 7.15)	2.92 (1.12 to 7.60)

IC: The proposed method for calculating pseudo-observations for interval censored data

CIP: Cumulative incidence proportion

CI: Confidence interval

## Discussion

With the methods proposed in this article, we have provided a way to calculate pseudo-observations and hence perform regression modeling in data consisting of both right and interval censored data on an event of interest which is subject to competing risks. We have shown by simulations that this method gives unbiased estimates of the cumulative incidence function in a realistic setting. Our proposed method also provides confidence intervals that have coverage probabilities close to the nomimal value. Our method is a further development of an approach for right censored competing risks data [12] and compared to the recently proposed method by Sabathé et al. [11] it requires relatively few parameters and does not require any analyst choices apart from determining the spline knots.

There are a number of considerations and assumptions for the parametric pseudo-observations for right censored data that also apply to the interval censored version. This concerns the assumption of independent censoring as well as the choice of number and positions of knots for the splines. For the interval censored data, we have imposed the additional assumption that the examination times are independent of the risk of the event of interest.

The methods that we propose in this article are not implemented in any standard software packages, but we have provided a publicly available Stata syntax example on GitHub [17].

A practical limitation of our method is that it is a very computationally intensive task to estimate the spline coefficients in each leave-one-out subsample of the dataset. On a standard laptop running Stata 16.1, we experienced a run-time for one sample of 250 observations of about 1h7m using 3 spline knots. Fortunately, this need only be done once for each study. This is also the reason for our limited number of repetitions in our simulation study.

Although we allow that the event of interest is either right or interval censored or a mix of both, we have only considered the case where the time of the competing event is exactly observed. If this is not the case and the competing event is also interval censored, the situation is far more complicated. This is unlikely to be the case when death is the only competing event but it could be relevant if other events can preclude the event of interest. Our proposed methods do not cover this situation and are not easily extended to do so.

A special case of interval censored data to which our methods do apply is known as *current status* data in which we only have one examination for each individual. One example of such data is information from a systematic population screening for a specific condition. For a non-congenital condition, a positive screening would provide information that the condition has occurred at some point prior to the screening but nothing more yielding long intervals that reflect the uncertainty of the exact occurrence time of the condition.

## Conclusion

In this article, we have shown how the previously proposed parametric pseudo-observations for right censored data can be extended to cover a setting with both right and interval censored data. Since interval censored data are almost inevitably subject to the competing risk of death, we have formulated the methods in an illness-death model that accommodates this circumstance. We have demonstrated through simulations that the proposed method performs well with no noteworthy bias and satisfactory coverage probabilities for estimating the cumulative incidence as well as absolute and relative associations with an exposure.

## Supplementary Information

**Additional file 1** Likelihood contributions for each individual trajectory.

## Data Availability

The simulated datasets used and analysed during the current study are available from the corresponding author on reasonable request. The ICD data that support the findings of this study are available from the Danish Pacemaker and ICD Register but restrictions apply to the availability of these data, which were used under license for the current study, and so are not publicly available. Data are however available from the authors upon reasonable request and with permission of the Danish Pacemaker and ICD Register.

## Abbreviations

ICD: Implantable cardioverter-defibrillator
CI: Confidence interval
empSE: Empirical standard error
modSE: Average model standard error
RMSE: Root mean squared error
