# Supplementary material for: Regression models for interval censored data using parametric pseudo-observations
Source: BMC Med Res Methodol. 2021 Feb 15;21:36. doi: 10.1186/s12874-021-01227-8 (PMC7883580; doi:10.1186/s12874-021-01227-8)
Supplement: Supplementary file 1 — Additional file 1 Likelihood contributions for each individual trajectory. [file 12874_2021_1227_MOESM1_ESM.pdf]

Appendix

This appendix provides a description of the likelihood contributions for each individual following one of the six possible trajectories.

Trajectory 1

If an individual has an exactly observed event of interest at time t_{1i} and is then right censored at time t_{2i}, the corresponding contribution to the likelihood function is

$$L_i = S(t_{1i})h_{01}(t_{1i})\frac{\exp(-H_{12}(t_{2i}))}{\exp(-H_{12}(t_{1i}))}.$$

Trajectory 2

If an individual has a negative examination at time l_{1i} and is then right censored at time t_{2i}, the contribution is

$$L_i = S(t_{2i}) + \int_{l_{1i}}^{t_{2i}} S(u)h_{01}(u)\frac{\exp(-H_{12}(t_{2i}))}{\exp(-H_{12}(u))}du.$$

This likelihood contribution also applies to individuals with right censoring of the event of interest, since this corresponds to the special case where $l_{1i} = t_{2i}$ and the integral is thus zero.

Trajectory 3

If an individual has an interval censored event of interest occurring between time l_{1i} and t_{1i} and is then censored at time t_{2i}, the contribution is

$$L_i = \int_{l_{1i}}^{t_{1i}} S(u)h_{01}(u)\frac{\exp(-H_{12}(t_{2i}))}{\exp(-H_{12}(u))}du.$$

Trajectory 4

If an individual has an exactly observed event of interest at time t_{1i} and then dies at time t_{2i}, the contribution is

$$L_i = S(t_{1i})h_{01}(t_{1i})\frac{\exp(-H_{12}(t_{2i}))}{\exp(-H_{12}(t_{1i}))}h_{12}(t_{2i}).$$

Trajectory 5

If an individual has a negative examination at time l_{1i} and then dies at time t_{2i}, the contribution is

$$L_i = S(t_{2i})h_{02}(t_{2i}) + \int_{l_{1i}}^{t_{2i}} S(u)h_{01}(u)\frac{\exp(-H_{12}(t_{2i}))}{\exp(-H_{12}(u))}h_{12}(t_{2i})du.$$

Again, this applies to individuals with right censoring of the event of interest.

Trajectory 6
If an individual has an interval censored event of interest occurring between time l_{1i} and t_{1i} and then dies at time t_{2i}, the contribution is

$$L_i = \int_{l_{1i}}^{t_{1i}} S(u) h_{01}(u) \frac{\exp(-H_{12}(t_{2i}))}{\exp(-H_{12}(u))} h_{12}(t_{2i}) du.$$

If we furthermore use the indicator, d_{2i}, for the competing event (exactly observed), we can write all likelihood contributions as one of the following three expressions.
